# Prosocial sharing with organizations after the COVID-19 pandemic: A longitudinal test of the role of motives for helping and time perspectives

**DOI:** 10.1371/journal.pone.0310511

**Published:** 2024-09-18

**Authors:** Iwona Nowakowska, Joanna Rajchert, Dorota Jasielska

**Affiliations:** Institute of Psychology, The Maria Grzegorzewska University, Institute of Psychology, Warsaw, Mazovia, Poland; Georg-August-Universität Göttingen: Georg-August-Universitat Gottingen, GERMANY

## Abstract

The time after the COVID-19 pandemic posed a threat to engagement in prosocial behaviors within charity organizations. In the current study, we intended to test how three motivational paths: affective empathy-based, life satisfaction-based, and perceived social support-based shaped the change in intentions to give money and time to charity organizations over a yearly period (right after revocation of the most essential anti-COVID-19 laws and at the same time, outbreak of the war in Ukraine in 2022, in May 2022 and after a year, in late April-early May 2023). We also tested how past negative and present hedonistic time perspectives–namely, those most robust in predicting emotional states ‐ shaped the changes in the abovementioned motivational paths and giving intentions. We conducted our longitudinal study on the general population of Poland (*N* = 566). We found that there has been a significant drop in the willingness to give time to organizations over the year after loosening the COVID-19 restrictions and the outbreak of war in Ukraine. We found that affective empathy helped sustain the intentions to give time to organizations, whereas past negative time perspective contributed to the decrease in such intentions. Our study suggests threats to organizations and highlights potential ways to encourage supporting them and caring for their volunteers’ well-being.

## Introduction

A prolonged cutoff from the social support networks during the COVID-19 pandemic had a twofold impact on prosociality [1]. It limited opportunities to act prosocially in the same ways as before [2]. It caused household economic struggles [3], threatening traditional in-person volunteering [4]. However, it also encouraged the emergence of novel forms of prosociality, such as virtual volunteering [5] and increased generosity based on “catastrophe compassion” [6]. Some people engaged prosocially due to more free time to be spent on leisure activities and the desire to reconnect with others and feel needed [1].

Little time had passed after the COVID-19 lockdowns when another crisis emerged—namely, the most significant influx of refugees since World War II. It happened due to the war in Ukraine that started in February 2022 [7]. It caused an unprecedented mobilization of citizens to react and support incoming people in need quickly [8, 9]. Humanitarian corridors were prepared immediately, and numerous solidarity initiatives emerged in response to the needs of refugees [10].

Charitable organizations significantly encouraged and sustained prosocial acts toward others and the environment during the COVID-19 pandemic [11] and the refugee crisis related to war in Ukraine [12]. However, does the tendency to help continue in the "post-COVID-19" world, as the pandemic has been a strong and unprecedented social situation for most people in Europe [13], and a full-scale war in one of the countries on the continent goes on?

In the current paper, we posed a research question: whether the willingness to donate money and time to organizations changed after the pandemic, and what motives affected the magnitude and direction of that change. We wanted to contribute to answering this question by conducting a yearly longitudinal study. We aimed to explore five predictors that can contribute to the change in giving intentions. First, we took into account three potential motivations to help as the predictors of intentions to give: (1) emotion-based care for others’ welfare (affective empathy); (2) satisfaction with life; (3) perceived social support–the first one of an altruistic/intrinsic kind, and two latter–of more egoistic/extrinsic type (see a theoretical framework on prosocial motivation [14] and qualitative insight into volunteer motivations during COVID-19 pandemic [15]).

Additionally, we pose a question of how these motives for prosociality are shaped by more general tendencies to think about past life events and experiences and the consequences of one’s own actions for one’s own hedonistic pleasure–namely, past negative and present hedonistic time perspectives. These time perspectives are the most robust predictors of emotional states [16]. These time perspectives also seem crucial to understanding motivations to help in the current post-COVID-19 times, as previous studies suggested that adverse life experiences may facilitate empathy and prosocial behaviors [17]. In contrast, present hedonism can be related to helping due to feeling satisfaction and immediate pleasure about giving to others during a crisis [18]. Moreover, time perspectives are amendable [19]; thus, finding out which of them should be targeted seems worthwhile in planning how to encourage prosociality in individuals.

Such an analysis has not been performed to date, and our study is unique in terms of its timing and geographical setting (Poland). It can be especially interesting given that Poland has been a leader in accepting Ukrainian refugees after the war outbreak [8]. Our study was performed in May 2022 and 2023, enabling us to capture the initial and delayed prosocial motivations with the refugee crisis in the background.

The pioneering nature of the project reveals in (1) the time-sensitivity and timeliness of the research problem; (2) the longitudinal character of the study, which enables us to go beyond correlational relationships; (3) the multi-predictor character of the studies, which will take into account several individual differences predicting prosociality.

The study can have an impact by adding to the knowledge regarding the patterns of tendencies for social mobilization/deterioration amid a crisis and afterward. It will also empirically test an integrative path model explaining prosociality patterns within an overarching theory of prosocial motivation [14] and the time perspective theory [19]. It is going to widen the growing body of studies on yet under-researched aspects of linkages between prosocial motivation and temporal orientations (for the theoretical framework, see [19] for available empirical data, e.g., [20, 21]). The model to be tested has not yet been assessed empirically, and it is meant to contribute to the theoretical discourse about prosocial motivation and time perspectives theory. Understanding the mechanisms of prosocial giving can help charitable organizations address prosociality crises in the future, for example, by tailoring the communication about the opportunities to support them or, in the case of volunteers’ recruitment, focusing recruitment and retention strategies on volunteers’ features relevant to prosocial intentions.

### Organizations during and after the pandemic–the role of prosocial sharing

The causes for which the charity organizations operate can be broadly described as the welfare of people and nature-related/environmental causes [22]. Helping by engaging with charitable organizations can be done by using financial resources to support others (prosocial spending) or by offering one’s time (volunteering). Interestingly, volunteering activities and donations are interrelated: the probability of charitable giving is higher in volunteers than non-volunteers, and the propensity to volunteer is higher in charitable donors than non-donors [23].

The COVID-19 pandemic and a consecutive lockdown have brought new ways in which people and organizations can help others [2, 11]. For example, local communities organized different forms of supporting people from vulnerable populations with food and medicine delivery. However, the patterns of the activity of volunteers or donors collaborating with them fluctuated [4, 11]. The subsequent crisis of Ukrainian refugee inflow showed that people tended to self-organize and were dissatisfied with how slowly charitable organizations operated in the first days of the war [8]. Nevertheless, organizations remain crucial actors in the mechanisms of formalized help provided to people in need during extraordinary times [24]. It leads us to pose questions about the prosocial intentions toward organizations after the crises occurred and the mechanisms that drive them. To date, no longitudinal data on that matter are available, which take into account not only the increase/decrease of prosocial tendencies but also the individual differences that are important in motivating to act prosocial.

As social support mobilization theory implies [25], in an immediate response to a crisis, people may be inclined to engage in helping. It was visible during the COVID-19 pandemic [18] and at the beginning of the Ukrainian refugee influx [8]. However, as the social mobilization patterns show [25], the deterioration phase should come over time, and consequently, the helping tendencies diminish. That is why we can expect that throughout the investigated period:

(H1) There is a decrease in the intentions to give money and time to organizations over time. However, what factors drive that change? We propose that to understand the patterns of changes in prosocial intentions, we need to consider individual differences that encourage helping [14] and the factors that may shape their usage in the decision-making process–time perspectives [19].

### Motives for helping

Reykowski and Smolenska [14] proposed a theoretical framework comprehensively addressing prosocial motivation. They distinguished three motives for helping: intrinsic, endocentric, and ipsocentric. The intrinsic motives concentrate on the needs of the other person. The helper focuses on the external world, precisely the person who needs help: the state, needs, and feelings of this person. Changing the other person’s situation is a reward, regardless of the other benefits for the person providing support. The endocentric motives concentrate on anticipating benefits for the helper’s self-esteem. The helper’s attention is focused on the self and its moral aspect. Finally, the ipsocentric motives stem from the need to gain personally or avoid loss.

Intrinsic motivations are generally more effective than extrinsic in sustaining activities, as they come from within a person [26] and not from the desire to obtain particular external outcomes, as it is for endocentric or ipsocentric motivations. That is why it could be supposed that the intrinsic reasons to help are responsible for the stability of prosocial behaviors over time.

We argue that during the COVID-19, people’s motivation for prosocial acts can be revealed in the willingness to (1) relieve empathic feelings towards others (which can be considered an intrinsic motive [27]), (2) sustain their positive feelings about themselves and their life (endocentric motive); (3) gain personally or avoid losses–by being part of a network of support exchange (ipsocentric motive).

For the intrinsic motive, empathy is one of the potential correlates of prosocial giving of money [28, 29] and time [30]. Empathy is the ability to understand and share another’s emotional state or context [31], thus encompasses affect congruence (affective empathy) and understanding of others’ emotions (cognitive empathy; [32]).

Extensive work by Batson and colleagues (e.g., for a review, see [33]) on the empathy-altruism hypothesis has shown that empathy promotes altruistic motivation, intending to increase the well-being of another person. Batson [34] defines empathy as an emotional response, oriented at other people, evoked by and congruent with the perceived welfare of an observed person in need. This definition resembles the affective and not the cognitive aspect of empathy, which is why we suppose that affective empathy specifically may promote giving to relieve the difficulties faced by another. For example, a person can understand the feelings of another person (cognitive empathy) but have no emotional reaction to these feelings (affective empathy), which could lead to actions that do not certainly alleviate the suffering of another person [35]. Empathy, as understood by Batson, connects with feelings of tenderness, compassion, and warmth [36].

Empathic feelings might have been relevant in the case of giving during the COVID-19 pandemic and the Ukrainian crisis, as people were directly or indirectly (e.g., through images in the media) witnessing the suffering of others. Studies have also shown that empathy relates to prosocial behaviors during the pandemic [37–39] and during the inflow of Ukrainian refugees [40]. Thus, the higher the empathy, the giving intentions should be higher and less prone to a decrease over time. We therefore hypothesize that:

(H2) There is a within-time positive correlation between affective empathy and intentions to give time and money, and affective empathy contributes to sustaining giving intentions over time.

For the endocentric motive for prosociality, there is a vast body of research regarding the linkages between well-being indicators, such as life satisfaction and prosociality (for recent meta-analyses, see [41, 42]. This link was also confirmed during the COVID-19 pandemic [43]. Helping others might be an adaptive mechanism that could have been used during the pandemic. Being cut off from the social network, people could have reacted to the limited opportunity to support others [1]. Prosocial behavior can also reinforce satisfaction with life [44], forming a reciprocal relationship and sustaining prosocial intentions over time. Therefore, we hypothesize that:

(H3) There is a within-time positive correlation between satisfaction with life and intentions to give time and money, and satisfaction with life contributes to sustaining giving intentions over time.

The perceived social support as a predictor of prosociality can be considered a mechanism for calculating the costs and rewards of prosociality, resembling the ipsocentric view of prosocial behaviors. Providing support to others can be viewed as rational behavior, especially in times of crisis or deficits [45], and mobilization of support in this case is a typical social response [25]. During the pandemic, social support was exchanged [46, 47], producing norms of reciprocity. Grassroots initiatives answered the social system’s needs (a need for solidarity in the face of a crisis–first, the lockdowns, then, the economic crisis to come). An example of such a grassroots initiative during COVID-19 was the network of numerous Internet-based groups exchanging neighbor support [18].

Some studies suggest that perceived social support during the pandemic strongly predicted prosocial behaviors across countries [43]. It might be because individual contacts play a role in social exchange [48]. Human needs are satisfied by others, but the interactions must involve mutual costs and rewards. The grassroots initiatives and helpful relationships formed new social structures, which should be sustained as long as people obtain rewards for costs borne. We can assume in this case that the reward, and at the same time, the antecedent of bearing further costs (acting prosocially toward others), is the perceived social support. Therefore, we hypothesize that:

(H4) There is a within-time positive correlation between perceived social support and intentions to give time and money, and perceived social support contributes to sustaining giving intentions over time.

### Time perspectives and their role in motives for prosocial sharing

To further understand people’s behaviors in the context of a social crisis such as the COVID-19 pandemic or the refugee crisis, we argue that it is worthwhile to include to what degree people are anchored in their negative past experiences and how they can seek pleasure and "catch" what the present brings. These aspects can shape worldviews and actual behaviors by influencing distress responses and cognitions in the face of difficulties [18, 19, 49, 50]).

These tendencies belong to the time perspectives. Time perspectives [51] are a tendency to assign experiences to timeframes of the past, present, or future. When used habitually, time perspectives can become a relatively stable individual difference [19, 51]. Traditionally, time perspectives are distinguished into the past negative (a discouraged view of the past), past positive (a nostalgic view of the past), present fatalistic (a conviction that one should follow the flow of experiences as it cannot be changed), present hedonistic (connected to deriving pleasure and seeking sensation in the present moment) and future (concern about what is yet to come, a tendency to plan and work to achieve the desired effect, despite the costs [51]). Literature suggests that time perspectives–especially past negative and present hedonistic–relate to the intrinsic, endo-, and ipsocentric aspects of helping and thus may help understand the mechanisms responsible for sustaining helping behaviors over time. Importantly, these two time perspectives are the most robust in predicting current emotional states [16]. They were also proven to be related to emotional and behavioral responses during the first phases of the COVID-19 crisis–past negative time perspective was a correlate of loneliness during lockdown [50], and present hedonistic–of giving resources to others during the first social distancing period [18].

Data on the links between different time perspectives and empathy is not supported by clear theoretical or empirical evidence. Studies are available regarding the other three time perspectives and life satisfaction [52, 53] and perceived social support [50, 54]; however, the results regarding the relationships between them are less clear in terms of theoretical clarification, which could pertain to further prosocial intentions.

#### Past negative time perspective, motivations to help, and prosocial intentions

We argue that past negative time perspective is crucial for our models, given that past experiences, especially adverse ones, may lead to poorer mental health outcomes [55] and shape worldviews and attitudes [56]. Therefore, understanding how negatively a person is focusing on his or her past can help understand the motivations for prosociality and the prosocial intentions themselves.

For the evidence supporting the predictive role of this time perspective for motivations for prosociality, the number of lifetime traumas is correlated with empathy [57], especially for people who have experienced similar traumatic events [58]. Negative cognitions about the self and the world are commonly observed among survivors of trauma [59]. We can thus presume that:

(H5) Past negative time perspective, as a result of negative experiences from the past, is positively correlated within-time to affective empathy, and contributes to its sustaining over time.

Past negative time perspective is also related negatively to satisfaction with life, as it promotes a discouraged view of the past that hinders well-being [60, 61]. It is also a predictor of anticipation of negative moods [16]. We hypothesize that:

(H6) Past negative time perspective is negatively correlated within-time to satisfaction with life, and contributes to its decrease over time.

As for the idea of helping as an exchange of social support with others, higher concentration on the negative past can be related negatively to perceived social support [50, 54]. It is because the past negative time perspective is related to higher conflict with family and low support from them. We therefore hypothesize that:

(H7) Past negative time perspective negatively correlates within-time to perceived social support, and contributes to its decrease over time.

Finally, for prosocial giving intentions, it has been proven that people who experienced trauma in the past can be inclined to act prosocial [57]. Altruistic tendencies might be observed as a psychological response to being a victim of adverse life events [62]. As argued above, people with traumatic and adverse experiences may have the most reasons to have a negative outlook on their past and, as a result, past negative time perspective. That is why we suppose that:

(H8) Past negative time perspective positively correlates within-time to intentions to give money and time to organizations and contributes to its sustaining over time.

#### Present hedonistic time perspective, motivations to help, and prosocial intentions

The present hedonistic time perspective pertains to how a person is ready to engage in the here and now to obtain pleasure from their own actions [51]. It positively correlates with empathy [63]. Empathy can be understood as a "here and now" emotion in response to the currently observed state of another person (congruent with the idea about the positive role of the present hedonistic time perspective in helping [19]). That is why we hypothesize that:

(H9) Present hedonistic time perspective positively correlates within time to affective empathy and contributes to its sustaining over time.

Present hedonistic time perspective is positively related to satisfaction with life [60], as it drives the desire to seek pleasure and experience joy in the here and now [13]. It also mediated the relationship between the perceived adverse impact of COVID-19 on well- and ill-being [64]. We thus suppose that:

(H10) Present hedonistic time perspective is positively correlated within time to satisfaction with life and contributes to its sustaining over time.

The present hedonistic time perspective is connected to having large support networks, consisting primarily of acquaintances and friends [54]. Thus, if people with a high present hedonistic time perspective were cut off from the sources of their support during the social distancing period, their heightened tendency to act prosocial might have been a compensatory strategy [18]. We thus hypothesize that:

(H11) Present hedonistic time perspective positively correlates within-time to perceived social support, and contributes to its sustaining over time.

Finally, this time perspective has been proven crucial in help provision during the first weeks of the COVID-19 pandemic [18], presumably because it drives to action over inaction in the case of emergencies and states of need. We suppose that:

(H12) Present hedonistic time perspective is positively correlated within time to the intention to give money and time to organizations and contributes to its sustaining over time.

In summary, in the current study, we will test past negative and present hedonistic time perspectives as the antecedents of pathways to prosociality: empathy, satisfaction with life, perceived social support, and prosocial giving intentions.

### Current study

In our study, we aim to test how the intentions to give to organizations are shaped throughout time, from right after the revocation of the essential pandemic-related regulations and an outbreak of war in Ukraine (May 2022) to a year afterward (late April-early May 2023). Firstly, we test whether there is a drop or increase in the intention to give money and time to organizations over time. Next, we test how three paths of potential motivations: empathy, satisfaction with life, and perceived social support, shape the prosocial giving intentions of coins and time (using Dictator Game-based items, in which participants decided how much coins or time they are to give to a particular organization). Dictator Game is a typical measurement for giving propensity and has been used in similar research [65]). We supposed that all potential motivations positively predict prosocial intentions within waves and over time. Secondly, we wish to test how time perspectives reported to be conceptually and empirically related to the individual differences shaping prosocial motivations: present hedonistic and past negative shape these motivational paths and prosocial intentions over time.

Our approach focuses not on real-life behaviors but on behavioral intentions. We will examine how individual differences shape the generalized intentions to help and identify the general population-level mechanism that may encourage giving to organizations as vital charity actors. It is the first study to examine these specific mechanics and broaden the knowledge of prosocial motivations, time perspectives, and social support mobilization patterns.

## Materials and methods

### Participants

*N* = 977 people participated in the first wave of study and were recruited to reflect the structure of the Polish population in terms of gender, age, size of place of residence, and education, referring to data from Statistics Poland [66], however, out of them, *N* = 566 participated in the second wave, constituting the final sample (the panel attrition was 42%). It consisted of 286 females and 280 males, aged 18–65, *M*_*age*_ = 44.86; *SD*_*age*_ = 12.47. For place of residence, 259 people (45.8%) were village inhabitants, 135 (23.9%) lived in a town with up to 50,000 inhabitants, 29 (5.1%) in a town with between 50,001 to 100,000 inhabitants, 84 (14.8%) in a town with between 100,001 to 500,000 inhabitants, 59 (10.4%) in a town with over 500,000 inhabitants. For education, 37 people (6.5%) finished primary school, 168 (29.7%) vocational education, 194 (34.3%) high school, 44 (7.8%) Bachelor’s degree, 123 (21.7%) Master’s degree.

### Procedure

The study was done by a specialized research panel–ReaktorOpinii™ and performed online. The registered users of the panel took part. The first wave of the study took place between 4^th^ and 10^th^ May 2022 –a month after an extensive loosening of the COVID-19 restrictions, namely revoking the obligation to wear protective masks in public spaces (with medical facilities as the exception. It was considered a crucial step in the "return to the normality" phase. Moreover, it was a period ca. two months after a wave of refugees from Ukraine started to come to Poland, requiring support from the organizations functioning in the country and individual citizens [67]. The study’s second wave occurred between 20^th^ April and 4^th^ May 2023, approximately a year after the first wave was conducted.

Before both waves, the participants were informed that the study’s goal was to check how people decide to support other people or organizations depending on their traits, quality of relationships with others, and everyday feelings and that questions may concern personal experiences and views regarding the COVID-19 pandemic. All participants provided informed consent online before filling out the questionnaires by accepting the terms of the study, which were presented to them in written form on the first screen of the survey. The study was anonymous; no consequences were applied due to withdrawal from participation. The panel remunerated participants who finished any of the waves with points later exchangeable into money. The Maria Grzegorzewska University Research Ethics Committee approved the study protocol and materials, approval number 75/2022.

### Measures

**Time perspectives** were measured with the Zimbardo Time Perspective Inventory (ZTPI [51]; Polish version [68]). It is a self-report, 56-item scale constituted of five subscales: Past Positive (9 items), Past Negative (10 items), Present Hedonistic (15 items), Present Fatalistic (9 items), and Future (13 items). Sample item is: *Spending what I earn on pleasures today is better than saving for tomorrow’s security*. Participants marked their responses from 1 –very untrue to 5 –very true. Results in subscales were computed as means of items constituting the subscales. Results in subscales were computed as means of items constituting the subscales. Two subscales of interest were the Past Negative (PastN) and Present Hedonistic (PresentH). Cronbach’s α were: for Past Negative: .86 for the first wave, .87 for the second wave, for Present Hedonistic: .80 for the first wave, .82 for the second wave.

**Empathy** was measured with The Basic Empathy Scale for Adults [69]; Polish version: [70]). It is a self-report 20-item scale consisting of 2 subscales: Affective and Cognitive Empathy. Sample item is; *I don’t become sad when I see other people crying*. Participants marked their answers on a scale from 1 –*strongly disagree* to 5 –*strongly agree*. Only the Affective Empathy (AffEmp) subscale was of interest. The results were computed as means of items constituting the subscale. Cronbach’s α for it was .83 for both study waves.

**Satisfaction with life** (Satisfaction) was measured with the Satisfaction with Life Scale (SWLS [71]; Polish version: [72]). It is a self-report, unidimensional, 5-item questionnaire. Sample item: *So far*, *I have gotten the important things I want in life*. Participants marked their answers on a scale from 1 –*strongly disagree* to 7 –*strongly agree*. The global result was computed as the mean of items. Cronbach’s α for both waves of the study was .91.

**Perceived social support** (Support) was measured with four Berlin Social Support Scales ([73]; Polish version: [74]). The questionnaire has a self-report character. The following scales were used: Perceived Available Support (Emotional, PAS-E, four items, and Instrumental, PAS-I, four items); Need for Support (NFS, four items); and Support Seeking (SS, five items). Sample items are: *Whenever I am not feeling well*, *other people show me that they are fond of me*, *Whenever I am down*, *I look for someone to cheer me up again*. Participants answered on a scale from 1 –*totally untrue* to 4 –*totally true*. Results in subscales were computed as means of items constituting the subscales. Cronbach’s αs were: for Perceived Available Support: Emotional: .86 for the first wave, .88 for the second wave, for Perceived Available Support: Instrumental: .93 for the first wave, .94 for the second wave, Need for Support: .67 for the first wave, .68 for the second wave, Support Seeking: .90 for the first wave, .89 for the second wave.

#### Giving coins to organizations

The willingness to give coins/money to organizations (GM) was measured with Dictator Game-inspired items based on an idea by Van de Groep and colleagues [65]. The instruction stated: *Imagine that you have 10 coins at your disposal*. *These coins are very valuable to you*, *but also to other people*. *You can keep these coins for yourself or donate some or all to support someone or an institution from the list below*. *Decide how many coins you would donate to each of these persons/institutions as a form of support*. *You can give a maximum of 10 coins*. *Each time*, *decide as if you had 10 coins at your disposal*. The targets were a local organization operating for better life quality in one’s neighborhood, a local organization operating for pro-environmental issues in one’s neighborhood, a global organization operating for better life quality in the world, and a global organization operating for pro-environmental issues worldwide. For the analyses, the indicator of the propensity to give was constituted as a latent variable loaded by particular items.

#### Giving time to organizations

The willingness to devote time to organizations (GT) was measured with Dictator Game-inspired items based on an idea by Van de Groep and colleagues [65]. The instruction stated: *Imagine that you have 100% of your free time in the week at your disposal*. *Think that you can spend it on freely chosen activities*. *You can devote part or all of your free time to support various people/organizations in need*. *Decide what percentage of your free time you would devote to supporting each of the people/organizations in need listed below*. *You can devote up to 100% of your free time to each of them*. *Each time*, *decide as if you had 100% of your free time at your disposal*. The list of targets was the same as for giving money. For the analyses, the indicator of the propensity to give time was constituted as a latent variable loaded by particular items.

### Analytic strategy

First, we tested for systematic missingness of the data between the two waves. We compared participants who completed two waves with those who participated only in the first wave on all study variables. Significant differences between these groups would indicate variables correlating with the missingness and should be included in the model as auxiliary variables [75]. Adding auxiliary variables in the analysis increases the probability of the assumption of missing at random (MAR). Next, the data were analyzed with zero-order Pearson correlations.

Additionally, to test for the stability of the constructs, we used paired samples *t*-tests. We only expected changes over time in giving intentions (H1). For this analysis, measures of Support and Giving (GM, GT) were averaged, and mean indices of Support, GT, and GM were created. Finally, hypotheses were tested with a cross-lagged panel model using Structural Equation Modeling (SEM) and AMOS/SPSS software. It tested H2-H12 about sustaining/decrease of particular tendencies over time.

The theoretical model included data from two points in time and tested (1) the relationship between variables within each time-point (wave), (2) relationships between the same variables measured twice–autoregressive effects, and (3) the relationship between wave 1 predictors and wave 2 predicted variables–cross-lagged effects. Autoregressive effects test the stability of the constructs over time. The within-time correlations account for the situation-specific effects, and the cross-lagged effects test the effects of T1 variables on T2 variables, controlling for the autoregressive effects. In doing this, we tested H2-H12 regarding the within-time correlations.

The model included seven variables within each wave. There were three latent variables in the model: giving coins/money (GM) and giving time (GT) to organizations (each variable loaded by four observed variables measuring intentions to give money or time to (1) a local organization operating for better life quality in one’s neighborhood, (2) a local organization operating for pro-environmental issues in one’s neighborhood, (3) a global organization operating for better life quality in the world, (4) a global organization operating for pro-environmental issues in the world), and Support (loaded by Perceived Available Support–Emotional and Instrumental, Need for Support and Support Seeking). Effects (standardized regression weights) of latent variables on indicator variables in T1 and T2 are presented in S1 Table.

**Fig 1:**
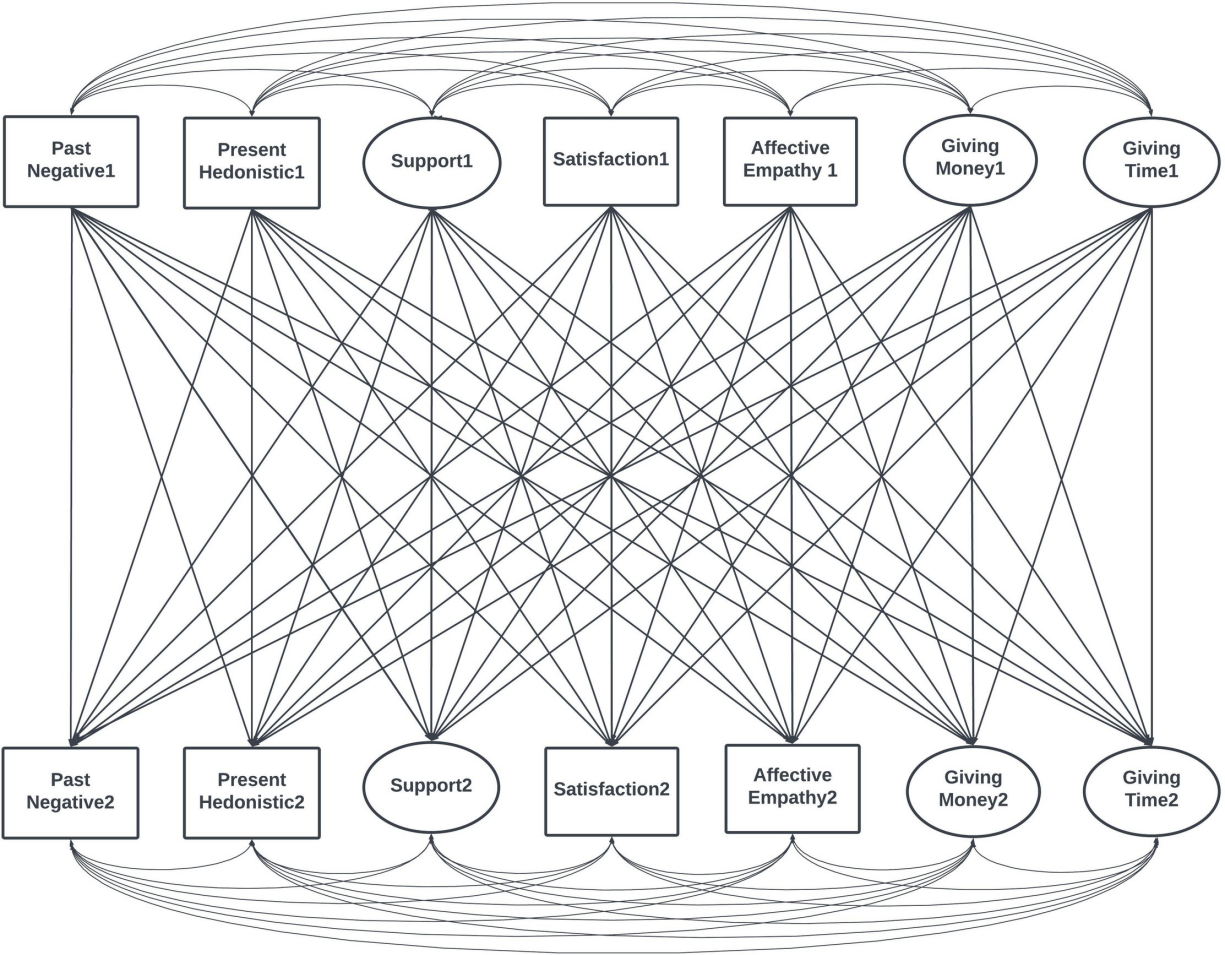
The theoretical cross-lagged panel model of giving time and money to organizations. Latent variables illustrated by ellipses are loaded by measured variables, which were not presented for clarity. Also, correlations of error terms were not presented but were included (for description, see the analytical strategy paragraph).

The observed variables in the model included Affective Empathy, Satisfaction (Satisfaction with Life), and Past Negative and Present Hedonistic time perspectives. We also correlated the errors of the same observed variables over time to account for indicator-specific effects. These correlations are shown in S2 Table. The entire theoretical model is presented in Fig 1.

All analyses were performed in IBM SPSS 29.0.0.0 [76] and IBM AMOS 28 [77].

### Note on data

The analysis presented here is part of a larger project. The analysis is original and has not been published before. Other texts based on the database that are prepared regard different research questions and dependent variables and are based on different theoretical models and estimations. None of them have been published yet in any form. The data can be accessed at https://osf.io/se6tg/

## Results

### Panel attrition

We conducted χ^2^ tests and *t*-tests to test for the differences in demographic variables and observed measures among participants who completed only the first measurement point (*N* = 411, 42%) or both measurement points (*n* = 566, 58%). This way, we could verify whether data were missing completely at random (MCAR) or the data were missing at random (MAR). Results regarding demographic characteristics indicated that participants who dropped from wave 2 were younger, *M* = 38.85, *SD* = 12.44, than those who finished the study, *M* = 44.86, *SD* = 12.46, *t* (1, 975) = -7.44, *p* < .001. However, there were no differences concerning gender, χ^2^ = .076, *p* = .782, and education, χ^2^ = 7.12, *p* = .212 among those two groups. Thus, we suspected that missingness could be related to age [78].

Furthermore, participants who completed only wave 1 were higher on GM, *M* = 1.75, *SD* = 2.23, and GT, *M* = 24.11, *SD* = 25.26 than participants who completed both waves [GM: *M* = 1.27, *SE* = 1.82, *t* (1, 975) = 3.56, *p* < .001; GT: *M* = 20.23, *SD* = 22.80, *t* (1, 975) = 2.47, *p* = .007]. Also, Seeking Support (SS) and Present Hedonistic (PresentH) time perspective were higher (*M*_SS_ = 2.81, *SD*; *M*_PresentH_ = 3.42, *SD* = 0.50) among wave 1 participants compared to participants of both waves [*M*_SS_ = 2.67, *SD* = 0.71; *t* (1, 975) = 3.21, *p* = .001; *M*_PresentH_ = 3.30, *SD* = 0.49; *t* (1, 975) = 3.67, *p* < .001].

Since age was the main cause of missingness, variables that differ between participants who completed both waves and those who dropped out should be related to age. Results showed that age was unrelated to giving intentions. However, it was negatively associated with Support Seeking and Need for Support. Furthermore, younger participants were higher on Past Negative and Present Hedonistic time perspectives. Correlation indices are presented in S3 Table. In such cases, age should be treated as an auxiliary variable and be added to the model estimated with full information maximum likelihood [75, 79]. Thus, in line with Enders’s [75] suggestions, we added age to the model using the saturated correlates approach.

### Bivariate correlations and differences between observed variables

Results of zero-order Pearson correlations between intentions to give coins/money (GM) to organizations at wave 1 and wave 2 are presented in S4 Table along with an analogous S5 Table regarding intentions to give time (GT). Giving intentions to different organizations were strongly correlated within each wave, both for giving time and giving coins (r’s > .80). The pattern of correlations between variables was very similar at both waves, which speaks for measurement invariance across time. Nevertheless, giving money and time were less strongly correlated (*p*’s below .50 for wave 1 and below .06 for wave 2), so it was justified to create separate latent variables of giving money (GM) and giving time (GT) at both measurement times loaded by observed giving intentions.

Results of zero-order Pearson correlations between wave 1 affective empathy, social support, and satisfaction with life and wave 1 and 2 giving intentions are presented in S6 Table, whereas analogous data for wave 2 affective empathy, social support, and satisfaction with life and wave 1 and 2 giving intentions are presented in S7 Table.

As for the relationship of predictors to giving intentions, Affective Empathy was positively associated with almost all indices of giving at both waves. Moreover, all indices of Support at both waves were positively associated with giving intentions. Also, Satisfaction 1 and 2 were positively associated with most of the giving indices at both waves. Nevertheless, only a few correlations were significant in the case of time perspectives and giving intentions. The pattern of correlations was very similar in both waves.

We also tested whether study variables were associated with demographic variables, gender, age, and education (coded as "not finished higher education (college/university)" vs. "finished higher education") in participants who completed both waves. Results showed that older participants were higher on Past Negative TP. Finishing higher education was related to more satisfaction with life and more intentions to give money to organizations (only wave 2). Furthermore, women scored higher than men in Affective Empathy, Support indices, Past Negative time perspective, and both giving intentions (GM and GT). Coefficients are presented in S8 Table.

Next, we conducted *t*-tests to test for differences between study variables over time and test H1. Regarding social support indices GM and GT, means were calculated based on observed indicators. The only significant change was indicated for GT; participants intended to give less time in wave 2 than in wave 1 (which confirmed H1 for GT and disconfirmed it for GM). These results initially supported the assumption regarding the temporal stability of the trait constructs. The results of these tests are presented in Table 1.

**Table 1:** Means and SD for study variables in waves 1 and 2 with results of difference tests.

Variables	Wave 1		Wave 2		t	p	d
	*M*	*SD*	*M*	*SD*			
GM	1.28	1.82	1.17	1.78	1.24	.217	0.052
GT	20.23	22.80	16.40	20.84	3.91	< .001	0.164
Social Support (mean)	2.91	0.56	2.92	0.57	-0.53	.595	-0.022
PAS^a^ Emotional	3.09	.66	3.12	0.70	-1.17	.241	-0.049
PAS^a^ Instrumental	2.74	.62	3.17	0.79	-1.05	.294	-0.044
Need for Support	3.14	.75	2.74	0.63	0.27	.788	0.011
Support Seeking	2.67	.71	2.66	0.74	0.48	.630	0.020
Affective Empathy	3.34	.61	3.33	0.60	0.54	.587	0.023
Satisfaction with Life	3.88	1.30	3.90	1.34	-0.77	.443	-0.032
Past Negative TP^b^	3.31	0.72	3.28	0.76	1.55	.122	0.065
Present Hedonistic TP^b^	3.31	0.50	3.28	0.52	1.58	.114	0.067

^a^PAS–Perceived Available Support.

^b^TP–Time Perspective.

### The cross-lagged model

The theoretical model was tested with covariance-based SEM using the full information Maximum Likelihood method to estimate discrepancy. The model did fit the data well (*χ*^2^(349) = 854.21, *p* < .001; RMSEA = .050, 90% CI .046; .055; NFI = .955; CFI = .973 [80]). The standardized lagged effects (standardized regression weights) in the cross-lagged panel model are presented in S9 Table.

**Fig 2:**
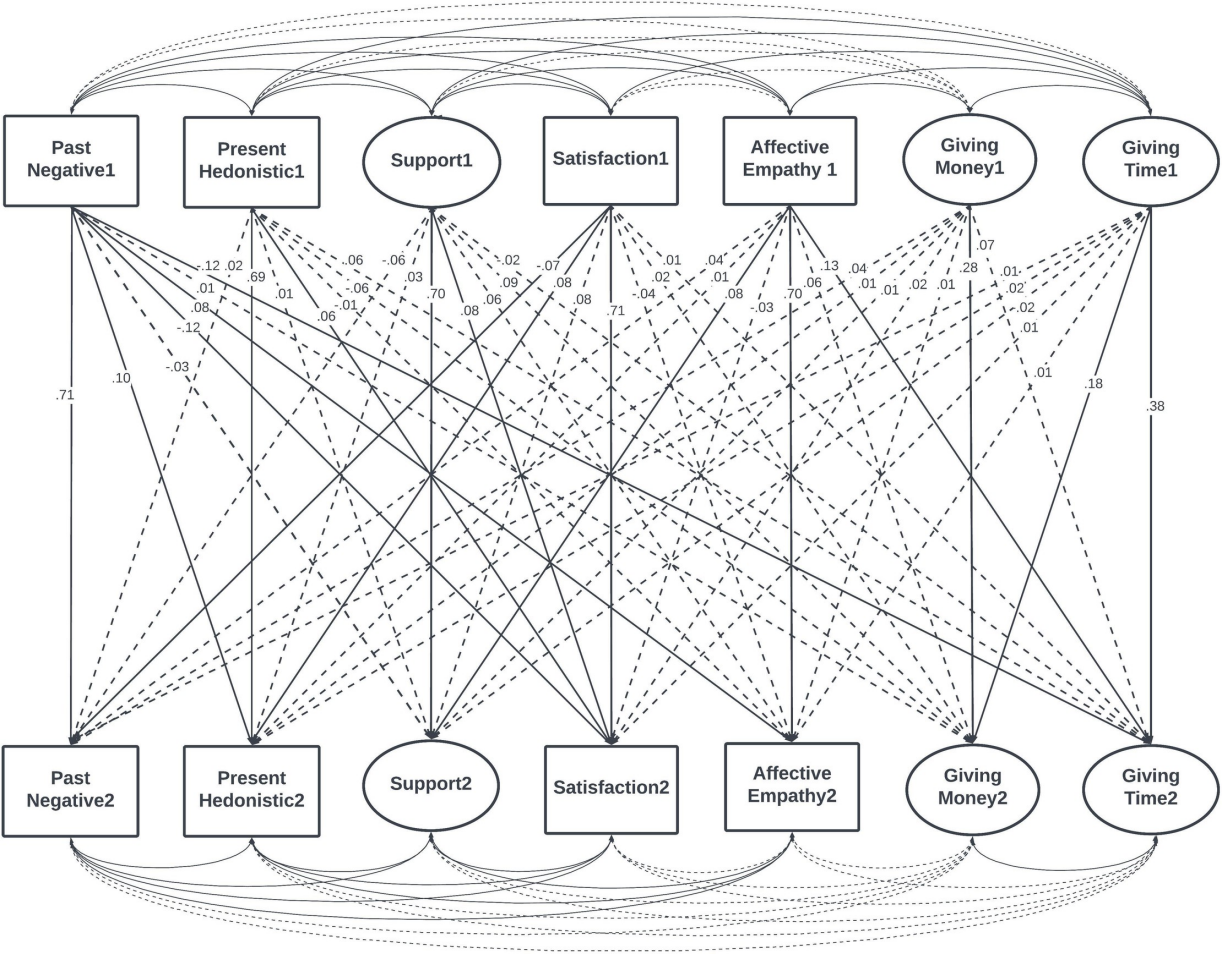
The empirical cross-lagged panel model of giving time and money to organizations. Solid lines represent significant paths and relationships. Only standardized regression weights are presented.

The final model is presented in Fig 2.

Because the formulation of the questions regarding giving intentions was very similar, some error terms were correlated within the same wave. Also, error terms of Need for Support and Support Seeking within waves were correlated in the model, as those constructs are closely associated. The correlations for age (auxiliary variable) and error terms of indicators are presented in SF S10 Table.

### The autoregressive effects

Each T1 variable in the model significantly affected the same T2 variable. As expected, autoregressive effects were positive and high for constructs representing traits. These results confirm the good stability of constructs over time. However, autoregressive effects for behavioral intentions of giving money and time were low for giving money and moderate for giving time, which indicates that behaviors are more dependent on the time of measurement.

### The within-wave relationships

Table 2 shows the correlations between study variables in wave 1 in the panel cross-lagged model, whereas Table 3 shows the analogous data for wave 2.

**Table 2:** Correlations between study variables within wave 1 in the panel cross-lagged model.

Variables	GT		AffEmp		Support		Satisfaction		PastN		PresH	
	*r*	*p*	*r*	*p*	*r*	*p*	*r*	*p*	*r*	*p*	*r*	*p*
GM	.59	< .001	.18	< .001	.07	.146	.06	.200	.03	.418	.001	.988
GT	-		.15	< .001	.15	.002	.14	.001	.04	.307	.10	.024
AffEmp			-	< .001	.19	< .001	.02	.697	.21	< .001	.17	< .001
Support					-		.51	< .001	-.29	< .001	.27	< .001
Satisfaction							-		-.52	< .001	.22	< .001
PastN									-		.17	< .001

**Table 3:** Correlations between study variables within wave 2 in the panel cross-lagged model.

Variables	GT		Empathy		Support		Satisfaction		PastN		PresH	
	*r*	*p*	*r*	*p*	*r*	*p*	*r*	*p*	*r*	*p*	*r*	*p*
GM	.58	< .001	-.02	.579	-.06	.187	.05	.231	.05	.270	.04	.358
GT	-		-.03	.486	-.01	.786	.02	.680	.01	.817	.009	.826
Empathy			-		.10	.034	-.08	.059	.28	< .001	.11	.013
Support					-		.20	< .001	-.16	< .001	.13	.008
Satisfaction							-		-.25	< .001	.20	< .001
PastN									-		.20	< .001

Within wave 1, intentions to give money were positively associated with giving time. Giving money was related positively to Affective Empathy (confirming H2 regarding within-time correlations) but not to other variables (not confirming H3, H4, H8, and H12 regarding within-time correlations). On the other hand, giving time was related to all study variables besides Past Negative TP. Also, Affective Empathy was correlated with all study variables except satisfaction. Time perspectives were positively correlated and related to all constructs, except for the lack of a significant relationship between Past Negative and giving intentions and Present Hedonistic and giving money. Present Hedonistic TP was also positively associated with Satisfaction and Support, and Past Negative TP was negatively associated with these variables.

In the second wave, giving money was associated only with giving time. Both giving intentions were unrelated to other variables (not confirming H2-H4, H8, H12). This indicates that the time 2 trait constructs had no additional effect on giving intentions besides autoregressive and cross-lagged effects. However, all other relationships resembled those in wave 1.

### The cross-lagged effects and hypotheses testing

Results indicated that only Affective Empathy (positively) and Past Negative time perspective (negatively) affected later intentions to give time, but not money (partially confirming H2 and disconfirming H2, H3, H8, and H12). Earlier giving time positively affected subsequent giving money, but giving money did not affect subsequent giving time.

Concerning time perspectives’ effects on other constructs, we predicted that the Past Negative time perspective would have a positive lagged effect on Affective Empathy (H5), which was confirmed. We also predicted that the Past Negative time perspective would have a negative lagged effect on Satisfaction with Life and Perceived Social Support (H6, H7), which was confirmed only in the case of satisfaction with life (confirming H6, disconfirming H7). Results also showed that it positively affected wave 2 Present Hedonistic time perspective.

Hypotheses regarding the Present Hedonistic time perspective posited that it would positively relate to subsequent Affective Empathy (H9), Satisfaction with Life (H10), and Perceived Social Support (H11). Results showed that it positively influenced only wave 2 Satisfaction with Life (confirming H10 and disconfirming H11 and H12).

Satisfaction with Life at wave 1 also negatively affected the Past Negative time perspective at wave 2 and positively affected the Present Hedonistic time perspective at wave 2, so the effects of Satisfaction with Life and time perspectives were reciprocal.

It is worth noting that Affective Empathy at wave 1 positively affected Perceived Social Support at wave 2, and Perceived Social Support at wave 1 was positively related to Satisfaction with Life at wave 2.

## Discussion

Our longitudinal analysis confirmed our H1 about giving time, but not money, as there was only a significant drop in intentions to give time to organizations. It might be due to the change in social life throughout the study–people were returning to regular in-site work, leaving less space for extra-work activities such as devoting time to charity. Moreover, as the causes to be supported were less visible in 2023 than in 2022 (as the COVID-19 pandemic topics faded out and the wave of refugees from Ukraine was not of the same quantity as at the beginning of the war), people might not have felt the need to devote that much time to such causes, as is typical of social support dynamics [25]. Interestingly, the more a person intended to give time at wave 1, the less drop in giving money occurred a year afterward. Devoting time to charitable causes is perceived as a more significant emotional investment and as more virtuous than giving money [81]. In line with these notions, being more inclined to give time is more likely to relate to general intentions to give, whereas giving money does not imply a greater willingness to give time.

Partially congruent with H2, affective empathy was positively linked to giving intentions to organizations within time (but only at the first measurement) and sustaining the intentions to give time throughout the study.

Affective empathy occurs automatically and spontaneously and is based on sharing emotions with others and experiencing these emotions with them [69]. As it shows up regardless of will, it can be considered an intrinsic drive to help–and intrinsic motivation, according to literature, is the most successful in sustaining the propensity to act in a certain way [82]. Our results are congruent with this notion.

Apart from the cross-lagged effects of affective empathy, past negative time perspective had a significant negative effect on giving time. This occurred despite the lack of within-time relationships between this perspective and any time perspectives studied. Therefore, we did not confirm H8.

Past negative time perspective is typically related to negative feelings, such as loneliness [50] and depressive symptoms [83]. People with such negative feelings may have fewer mental resources to share with others and, therefore, engage less in prosocial behaviors—especially resource-consuming, such as devotion to free time.

Such cross-lagged effect was not observed for satisfaction with life (contrary to H3), perceived social support (contrary to H4), or present hedonistic time perspective (contrary to H12). For H3, there was only one within-time correlation between satisfaction with life and giving time at wave 1, which aligns with previous research [84, 85]. The lack of significant correlation for giving money can be explained by previous findings where neither happiness nor psychological well-being were related to donating to charity [86]. For the lack of significant relationship in the cross-lagged model, notably, it considered other crucial predictors that appeared more important than satisfaction with life. Furthermore, although a vast number of studies indicated the existence of a phenomenon called the glow of the goodwill [87], it was primarily observed in experimental conditions, where positive affect was induced prior to the assessment of prosocial behavior [88–90]. As some scholars argue, prosocial behavior can be driven by arousal that facilitates positive thoughts about others and a tendency to approach [91]. Therefore, as dispositional measures of well-being refer rather to a global evaluation of one’s life than to the assessment of the intensity of experienced emotions, it is possible that the link between satisfaction and giving was not significant in the current study due to insufficient arousal (comparing to experimental manipulation).

Moreover, a meta-analysis on the relationship between practicing various forms of kindness and well-being indicates that the effect is modest and might depend on other factors, such as the types of helpers and the characteristics of recipients [41]. In line with this implication, one study found that the association between happiness and prosocial orientation was significant and positive for subjects with high trust and negative for low trustors [92]. Hence, future studies should further explore this field and seek other potential moderators.

Similar to satisfaction with life, for perceived social support, we found that it was positively linked to intentions to give time to organizations at wave 1. The reason behind this might be that the people who receive more social support may be less capable of helping others with material resources (and that is why they need the support of others). Perceived social support may, however, encourage giving time through a mechanism of reciprocity–receiving something from others, one wants to give it back and has the social network to do it. It aligns with previous studies regarding perceived social support and prosociality [93] and the recent findings suggesting donors more generously share time than money [94]. Nevertheless, perceived social support did not contribute to changes in the giving intention patterns in the cross-lagged model.

We confirmed hypotheses H5-H7 and H9-H11 about the within-time correlations between the investigated time perspectives and motivational paths to prosociality. Nevertheless, for the cross-lagged model, we showed that past negative time perspective may promote automatic affective empathy, thus the reactions to others’ suffering (fully confirming H5). It might be due to the ability to recognize the distress of others based on one’s own experiences, which is in line with a study on trauma and compassionate behaviors [57]. Past negative time perspective also contributed to decreased satisfaction with life (fully confirming H6), which aligns with previous studies [61]. Nevertheless, it did not predict decreased perceived social support, disconfirming H7.

Cross-lagged panel model analysis indicated that the present hedonistic time perspective did not predict sustaining affective empathy (not confirming H9) or perceived social support (not confirming H11) over time. However, it predicted satisfaction with life over time (confirming H10). Additionally, satisfaction with life measured at wave 1 predicted the present hedonistic time perspective at wave 2. Although most previous studies focused on how time perspectives shape life satisfaction (e.g., [61, 95], our result is also congruent with knowledge about present hedonism. Given that time perspectives may be shaped by life experiences and psychological influences [19], our study contributes to the knowledge that addressing life satisfaction may result in a more hedonistic, pleasure-seeking attitude toward life, such as argued in the broaden-and-build theories of positive emotions [96].

### Strengths, limitations, and future research directions

Our study is pioneering in testing the personal factors that may lead to giving patterns to organizations in the post-COVID-19 world. It focuses on the most typical prosocial giving forms: donating money and time. We integrated several theories and empirical insights in a novel way to test a comprehensive model, leading from the individuals’ time perspectives through three paths of prosocial motivation to prosocial giving intentions. We employed a longitudinal design to gain insight into the patterns of the willingness to give over a specific time, which was unique in history (a year of loosening the pandemic-related restrictions and the year in which the war in Ukraine unfolded).

Despite these strengths, our study is not free of limitations. In the first wave, the structure of the sample was representative of the population in terms of age, gender, size of place of residence, and level of education. However, the inevitable dropout that occurred in the second wave compromised it. We also measured the dependent variable, considering only four generalized types of charitable organizations. Our measures of prosocial motivation did not consider satisfaction derived from helping as a specific activity or the decision-making processes, which is the element of assessing the costs and rewards of acting prosocial. These could be used in further studies, especially those involving actual behavior measurement.

Furthermore, we conducted the study employing a research panel. Thus, the recruited people were only the panel’s registered users. Our study was also performed at a specific time, and we do not have data from the period of COVID-19 lockdowns or from before the pandemic, which could have broadened the scope of our understanding of the investigated phenomena.

In future studies, researchers should continue to use longitudinal designs in studies on prosocial behaviors, involve more covariates and time points, and survey a broader sample than research panel users, maintaining the representativeness of a particular population. It is also worth exploring whether moderating or non-linear effects are relevant to predicting prosocial intentions or behavior patterns.

### Practical implications

Based on our findings, several recommendations regarding strategies to increase prosocial sharing can be made. First of all, the study has shown that the level of affective empathy allowed to predict who would be more likely to devote their time to others both right away and one year later. This disposition is a good indicator of a prolonged motivation to give time. Therefore, charitable organizations seeking long-term collaborators should focus on recruiting volunteers with high affective empathy. The benefits of hiring empathetic workers go beyond nonprofit institutions. There is evidence that willingness to act prosocially is a valuable asset in a workplace, positively impacting both givers and receivers [97]. Hence, affective empathy is worth assessing upon recruitment and further being developed during prosocial engagement.

We also found that willingness to give money for a prosocial cause is determined by intentions to devote time in the past, consistent with previous studies [23]. This implies that strategies to encourage financial donation may be particularly effective among people who have already given their time to the organization. Since these individuals are already motivated to contribute, they could also be encouraged to act as fundraising ambassadors. They could promote prosocial spending by modeling such behavior.

Finally, past negative time perspective can diminish willingness to give time. This result is in line with previous findings on links between depressive mood and reduced prosociality [98]. As the Past-Negative time perspective affects the interpretation of the present [61], subjects dealing with difficult memories might not have the emotional resources to devote time and energy to helping others. This finding has important implications for supervisors in charitable organizations and workplaces where willingness to help is vital. It stresses the necessity of caring for the mental health of volunteers and other-oriented employees. By ensuring that they receive help, support, and emphatic concern in times of crisis or work overload, managers can support them in diminishing the impact of negative time perspective (or trying to change it, as time perspectives are amenable–[19]). As a result, taking good care of employees’ and volunteers’ well-being will lead to higher engagement in prosocial sharing.

## Conclusions

Our two-wave longitudinal study confirmed the theoretical assumptions about empathy as a crucial aspect of prosocial intentions towards organizations. It confirms the notions about intrinsic motives to help as the most important ones [14] and empathy as a crucial driver of altruistic responses [34]. Moreover, we found that empathy is cross-sectionally related to past negative and future time perspectives, suggesting that people with negative past experiences but also directed at long-term goals have the propensity to react emotionally to others’ suffering. Finally, we found that there has been a significant drop in the willingness to give time to organizations over the year after loosening the COVID-19 restrictions and the outbreak of war in Ukraine. It shows a potential threat to organizations in the post-COVID time. Our study suggests that organizations could use emotion-based communications to encourage prosocial engagement and focus on the relevance of support to achieving long-term goals to attract future-oriented individuals. As people with past negative time perspective are inclined to help, it is crucial to pay attention to their well-being, compassion fatigue, and potential burnout, for example, by offering supervision for volunteers and caring for positive, secure relationships within an organization.

## Supporting information

S1 Table Effects (standardized regression weights) of latent variables on indicator variables in T1 and T2. All effects are significant, *p* < .001.
(DOCX)

S2 Table Correlations between residuals (errors) of indicator variables. **p < .001; *p < .05.
(DOCX)

S3 Table Correlations between demographic variables (age, gender, education) and observed study variables in wave 1 participants (*N* = 977). ** *p* < .001; * *p* < .05. Education was coded as 0 –high school/secondary or lower education level; 1 –higher education degree; gender was coded as 0 –women and 1 –men.
(DOCX)

S4 Table Pearson zero-order correlations between intentions to give money ‐ coins (GM) to organizations at time 1 and time 2. ** *p* < .001; * *p* < .05.
(DOCX)

S5 Table Pearson zero-order correlations between intentions to give time (GT) to organizations at time 1 and time 2. ** *p* < .001; * *p* < .05.
(DOCX)

S6 Table Pearson zero-order correlations between wave 1 empathy, perceived social support, satisfaction with life, time perspectives and intentions to give to money ‐ coins (M) and time (T) to organizations at time 1 and time 2. ** *p* < .001; * *p* < .05.
(DOCX)

S7 Table Pearson zero-order correlations between wave 2 empathy, perceived social support, satisfaction with life, time perspectives and intentions to give to money ‐ coins (M) and time (T) to organizations at time 1 and time 2. ** *p* < .001; * *p* < .05.
(DOCX)

S8 Table Correlations between demographic variables (age, gender, education) and observed study variables in participants of both waves (*N* = 566) in T1 and T2. ** *p* < .001; * *p* < .05.
(DOCX)

S9 Table The standardized lagged effects (standardized regression weights) in the cross-lagged panel model. (DOCX)

S10 Table Correlations between observed variables or residuals of observed variables and age as auxiliary variable in the panel cross-lagged model. ** *p* < .001; * *p* < .05.
(DOCX)
