# Supplementary material for: Prosocial sharing with organizations after the COVID-19 pandemic: A longitudinal test of the role of motives for helping and time perspectives
Source: PLoS One. 2024 Sep 18;19(9):e0310511. doi: 10.1371/journal.pone.0310511 (PMC11410197; doi:10.1371/journal.pone.0310511)
Supplement: S1 Table — All effects are significant, p < .001. (DOCX) [file pone.0310511.s001.docx]

**S1 Table.**

| **Latent** | **Observed Indicator** | ***β* T1** | ***β* T2** |
| --- | --- | --- | --- |
| GM | GM-LocalLife | .89 | .90 |
|  | GM-LocalEnv | .91 | .91 |
|  | GM-GlobalLife | .96 | .94 |
|  | GM-GlobalEnv | .98 | .97 |
| GT | GT-LocalLife | .85 | .93 |
|  | GT-LocalEnv | .91 | .94 |
|  | GT-GlobalLife | .89 | .89 |
|  | GT-GlobalEnv | .95 | .88 |
| Support | PAS-E | .89 | .95 |
|  | PAS-I | .89 | .89 |
|  | NFS | .42 | .39 |
|  | SS | .56 | .45 |
