# Supplementary material for: Prosocial sharing with organizations after the COVID-19 pandemic: A longitudinal test of the role of motives for helping and time perspectives
Source: PLoS One. 2024 Sep 18;19(9):e0310511. doi: 10.1371/journal.pone.0310511 (PMC11410197; doi:10.1371/journal.pone.0310511)
Supplement: S2 Table — **p < .001; *p < .05. (DOCX) [file pone.0310511.s002.docx]

**S2 Table.**

| **Variables** | | | ***r*** |
| --- | --- | --- | --- |
| GM-LocalLifeE1 | ⬄ | GM-LocalLifeE2 | .04 |
| GM-LocalEnvE1 | ⬄ | GM-LocalEnvE2 | .12** |
| GM-GlobalLifeE1 | ⬄ | GM-GlobalLifeE3 | .19** |
| GM-GlobalEnvE1 | ⬄ | GM-GlobalEnvE4 | .06 |
| GT-LocalLifeE1 | ⬄ | GT-LocalLifeE2 | .11* |
| GT-LocalEnvE1 | ⬄ | GT-LocalEnvE2 | .09 |
| GT-GlobalLifeE1 | ⬄ | GT-GlobalLifeE2 | .03 |
| GT-GlobalEnvE1 | ⬄ | GT-GlobalEnvE2 | .01 |
| PAS-EE1 | ⬄ | PAS-EE2 | .05 |
| PAS-IE1 | ⬄ | PAS-IE2 | .30** |
| NFSE1 | ⬄ | NFSE2 | .59** |
| SSE1 | ⬄ | SSE2 | .61** |
| GT-LocalLifeE1 | ⬄ | GT-LocalEnvE1 | .44** |
| GT-GlobalLifeE1 | ⬄ | GT-LocalEnvE1 | .21 |
| GM-LocalLifeE1 | ⬄ | GT-LocalLifeE1 | .11 |
| PAS-IE1 | ⬄ | NFSE1 | .15* |
| NFSE1 | ⬄ | SSE1 | .64** |
| NFSE1 | ⬄ | SSE2 | .52** |
| SSE1 | ⬄ | NFSE2 | .49** |
| GM-GlobalEnvE1 | ⬄ | GM-LocalLifeE1 | -.48** |
| GM-LocalLifeE1 | ⬄ | GM-LocalEnvE1 | .49** |
| SSE2 | ⬄ | NFSE2 | .64** |
| GM-LocalLifeE2 | ⬄ | GT-LocalLifeE2 | .14* |
| GM-LocalLifeE2 | ⬄ | GM-GlobalEnvE4 | -.65** |
| GM-LocalLifeE2 | ⬄ | GM-LocalEnvE2 | .47** |
| GT-LocalLifeE2 | ⬄ | GT-GlobalEnvE2 | -.43** |
| GT-GlobalLifeE2 | ⬄ | GT-GlobalEnvE2 | .36** |
| GM-GlobalLifeE3 | ⬄ | GT-GlobalLifeE2 | .33** |
| GM-LocalLifeE2 | ⬄ | GT-GlobalEnvE2 | -.41** |
| GT-LocalLifeE2 | ⬄ | GM-GlobalEnvE4 | -.58** |
| GM-LocalEnvE2 | ⬄ | GT-LocalEnvE2 | .34** |
