# Supplementary material for: Prosocial sharing with organizations after the COVID-19 pandemic: A longitudinal test of the role of motives for helping and time perspectives
Source: PLoS One. 2024 Sep 18;19(9):e0310511. doi: 10.1371/journal.pone.0310511 (PMC11410197; doi:10.1371/journal.pone.0310511)
Supplement: S3 Table — ** p < .001; * p < .05. Education was coded as 0 –high school/secondary or lower education level; 1 –higher education degree; gender was coded as 0 –women and 1 –men. (DOCX) [file pone.0310511.s003.docx]

**S3 Table.**

| **Variables** | **GM** | **GT** | **AffEmp** | **PAS-E** | **PAS-I** | **NFS** | **SS** | **Satisf** | **PastN** | **PresentH** |
| --- | --- | --- | --- | --- | --- | --- | --- | --- | --- | --- |
| Age | -.04 | -.02 | .01 | .04 | .02 | -.11** | -.10* | .05 | -.16** | -.12** |
| Education | .02 | .01 | .03 | .04 | .02 | -.01 | .00 | .02 | -.10* | -.08* |
| Gender | -.15** | -.20** | -.32** | -.13** | -.07* | -.18** | -.12** | .01 | -.11** | -.06 |
