# Supplementary material for: Prosocial sharing with organizations after the COVID-19 pandemic: A longitudinal test of the role of motives for helping and time perspectives
Source: PLoS One. 2024 Sep 18;19(9):e0310511. doi: 10.1371/journal.pone.0310511 (PMC11410197; doi:10.1371/journal.pone.0310511)
Supplement: S4 Table — ** p < .001; * p < .05. (DOCX) [file pone.0310511.s004.docx]

**S4 Table.**

| **Variables** | **LocalLifeM1** | **LocalEnvM1** | **GlobalLifeM1** | **GlobalEnvM1** | **LocalLifeM2** | **LocalEnvM2** | **GlobalLifeM2** | **GlobalEnvM2** |
| --- | --- | --- | --- | --- | --- | --- | --- | --- |
| LocalLifeM1 | 1 | .90** | .85** | .81** | .35** | .33** | .32** | .32** |
| LocalEnvM1 |  | 1 | .87** | .89** | .33** | .37** | .33** | .37** |
| GlobalLifeM1 |  |  | 1 | .94** | .33** | .34** | .39** | .40** |
| GlobalEnvM1 |  |  |  | 1 | .32** | .37** | .38** | .43** |
| LocalLifeM2 |  |  |  |  | 1 | .91** | .84** | .80** |
| LocalEnvM2 |  |  |  |  |  | 1 | .85** | .88** |
| GlobalLifeM2 |  |  |  |  |  |  | 1 | .92** |
| GlobalEnvM2 |  |  |  |  |  |  |  | 1 |
| ***M*** | 1.37 | 1.39 | 1.15 | 1.20 | 1.26 | 1.30 | 1.06 | 1.08 |
| ***SD*** | 1.83 | 2.01 | 1.87 | 1.93 | 1.91 | 1.99 | 1.79 | 1.82 |
