# Supplementary material for: Prosocial sharing with organizations after the COVID-19 pandemic: A longitudinal test of the role of motives for helping and time perspectives
Source: PLoS One. 2024 Sep 18;19(9):e0310511. doi: 10.1371/journal.pone.0310511 (PMC11410197; doi:10.1371/journal.pone.0310511)
Supplement: S5 Table — ** p < .001; * p < .05. (DOCX) [file pone.0310511.s005.docx]

**S5 Table.**

| **Variables** | **LocalLifeT1** | **LocalEnvT1** | **GlobalLifeT1** | **GlobaEnvT1** | **LocalLifeT2** | **LocalEnvT2** | **GlobalLifeT2** | **GlobalEnvT2** |
| --- | --- | --- | --- | --- | --- | --- | --- | --- |
| LocalLifeT1 | 1 | .87** | .80** | .80** | .41** | .37** | .36** | .38** |
| LocalEnvT1 |  | 1 | .80** | .87** | .39** | .38** | .37** | .36** |
| GlobalLifeT1 |  |  | 1 | .84** | .38** | .36** | .39** | .36** |
| GlobalEnvT1 |  |  |  | 1 | .38** | .36** | .40** | .39** |
| LocalLifeT2 |  |  |  |  | 1 | .87** | .82** | .75** |
| LocalEnvT2 |  |  |  |  |  | 1 | .83** | .81** |
| GlobalLifeT2 |  |  |  |  |  |  | 1 | .87** |
| GlobalEnvT2 |  |  |  |  |  |  |  | 1 |
| ***M*** | 21.19 | 21.77 | 18.92 | 19.05 | 17.75 | 17.48 | 14.91 | 15.48 |
| ***SD*** | 24.05 | 25.20 | 24.04 | 24.33 | 23.34 | 23.02 | 21.29 | 21.80 |
