# Supplementary material for: Prosocial sharing with organizations after the COVID-19 pandemic: A longitudinal test of the role of motives for helping and time perspectives
Source: PLoS One. 2024 Sep 18;19(9):e0310511. doi: 10.1371/journal.pone.0310511 (PMC11410197; doi:10.1371/journal.pone.0310511)
Supplement: S6 Table — ** p < .001; * p < .05. (DOCX) [file pone.0310511.s006.docx]

**S6 Table.**

| **Variables** | **AffEmp1** | **PAS-E1** | **PAS-I1** | **NFS1** | **SS1** | **Satisfaction1** | **PastN1** | **PresentH1** |
| --- | --- | --- | --- | --- | --- | --- | --- | --- |
| AffEmp1 | 1 | .18** | .14** | .33** | .32** | .02 | .21** | .17** |
| PAS-E1 |  | 1 | .80** | .38** | .50** | .46** | -.26** | .26** |
| PAS-I1 |  |  | 1 | .44** | .52** | .48** | -.30** | .20** |
| NFS1 |  |  |  | 1 | .72** | .09* | .14** | .17** |
| SS1 |  |  |  |  | 1 | .20** | .13* | .28** |
| Satisfaction1 |  |  |  |  |  | 1 | -.52** | .22** |
| PastN1 |  |  |  |  |  |  | 1 | .17** |
| PresentH1 |  |  |  |  |  |  |  | 1 |
| LocalLifeM1 | .17** | .08 | .02 | .12** | .12** | .07 | .02 | .01 |
| LocalEnvM1 | .15** | .09* | .01 | .08* | .11* | .04 | .02 | .01 |
| GlobalLifeM1 | .20** | .10* | .04 | .11* | .12 | .08 | .05 | .02 |
| GlobalEnvM1 | .16** | .09* | .01 | .08 | .08 | .04 | .03 | -.01 |
| LocalLifeM2 | .14* | .12* | .10* | .09* | .11* | .12* | -.05 | -.01 |
| LocalEnvM2 | .12* | .12* | .09* | .12* | .13* | .08 | -.04 | -.03 |
| GlobalLifeM2 | .18** | .13* | .09* | .12* | .16** | .10* | .01 | .02 |
| GlobalEnvM2 | .14** | .14* | .09* | .12* | .16** | .06 | .01 | .00 |
| LocalLifeT1 | .14** | .15** | .08* | .14** | .18** | .16** | .04 | .08* |
| LocalEnvT1 | .12* | .15** | .10* | .13* | .18** | .12* | .04 | .06 |
| GlobalLifeT1 | .15** | .14** | .08 | .13* | .18** | .15** | .02 | .10* |
| GlobalEnvT1 | .14** | .15** | .09* | .14** | .20** | .12* | .05 | .10* |
| LocalLifeT2 | .19** | .11* | .08* | .13* | .14** | .13* | -.06 | .11* |
| LocalEnvT2 | .14** | .10* | .08 | .11* | .14* | .12* | -.09* | .08 |
| GlobalLifeT2 | .21** | .11* | .07 | .13* | .18** | .14** | -.01 | .09* |
| GlobalEnvT2 | .17** | .11* | .07 | .11* | .17** | .12* | -.03 | .06 |
| ***M*** | 3.34 | 3.09 | 3.14 | 2.74 | 2.67 | 3.88 | 3.31 | 3.31 |
| ***SD*** | 0.61 | 0.66 | 0.75 | 0.62 | 0.71 | 1.30 | 0.72 | 0.50 |
