# Supplementary material for: Prosocial sharing with organizations after the COVID-19 pandemic: A longitudinal test of the role of motives for helping and time perspectives
Source: PLoS One. 2024 Sep 18;19(9):e0310511. doi: 10.1371/journal.pone.0310511 (PMC11410197; doi:10.1371/journal.pone.0310511)
Supplement: S7 Table — ** p < .001; * p < .05. (DOCX) [file pone.0310511.s007.docx]

**S7 Table.**

| **Variables** | **AffEmp2** | **PAS-E2** | **PAS-I2** | **NFS2** | **SS2** | **Satisfaction2** | **PastN2** | **PresentH2** |
| --- | --- | --- | --- | --- | --- | --- | --- | --- |
| AfEmp2 | 1 | .17** | .15** | .37** | .34** | -.08* | .31** | .16** |
| PAS-E2 |  | 1 | .84** | .37** | .43** | .47** | -.31** | .23** |
| PASI2 |  |  | 1 | .38** | .42** | .44** | -.28** | .20** |
| NFS2 |  |  |  | 1 | .71** | .05 | .19** | .20** |
| SS2 |  |  |  |  | 1 | .20** | .14** | .27** |
| Satisfaction2 |  |  |  |  |  | 1 | -.53** | .26** |
| PastN2 |  |  |  |  |  |  | 1 | .19** |
| PresentH2 |  |  |  |  |  |  |  | 1 |
| LocalLifeM1 | .13* | .02 | .03 | .10* | .10* | .08 | .05 | .03 |
| LocalEnvM1 | .13* | .02 | .02 | .05 | .07 | .05 | .06 | .03 |
| GlobalLifeM1 | .14* | .05 | .04 | .12* | .15** | .06 | .07 | .04 |
| GlobalEnvM1 | .12* | .04 | .04 | .08 | .09* | .04 | .07 | .03 |
| LocalLifeM2 | .09* | .05 | .06 | .06 | .07 | .13* | -.02 | .03 |
| LocalEnvM2 | .09* | .05 | .07 | .09* | .07 | .10* | .02 | .01 |
| GlobalLifeM2 | .12* | .07 | .08* | .11* | .13* | .11* | .05 | .05 |
| GlobalEnvM2 | .09* | .05 | .09* | .11* | .11* | .08 | .06 | .05 |
| LocalLifeT1 | .11* | .10* | .11* | .11* | .15** | .15** | .03 | .10 |
| LocalEnvT1 | .10* | .07 | .10* | .10* | .13* | .10* | .04 | .08 |
| GlobalLifeT1 | .10* | .07 | .08 | .11* | .13* | .12* | .05 | .12* |
| GlobalEnvT1 | .11* | .08* | .10* | .12* | .16** | .11* | .06 | .12* |
| LocalLifeT2 | .14** | .07 | .09* | .08 | .14* | .14* | -.03 | .11* |
| LocalEnvT2 | .07 | .07 | .09* | .06 | .10* | .11* | -.04 | .06 |
| GlobalLifeT2 | .13* | .10* | .12* | .15** | .16** | .11* | .02 | .11* |
| GlobalEnvT2 | .08 | .09* | .10* | .11* | .12* | .11* | -.002 | .06 |
| ***M*** | 3.33 | 3.12 | 3.17 | 2.74 | 2.66 | 3.28 | 3.47 | 3.33 |
| ***SD*** | 0.60 | 0.70 | 0.79 | 0.63 | 0.74 | 0.76 | 0.63 | 0.60 |
