# Supplementary material for: Prosocial sharing with organizations after the COVID-19 pandemic: A longitudinal test of the role of motives for helping and time perspectives
Source: PLoS One. 2024 Sep 18;19(9):e0310511. doi: 10.1371/journal.pone.0310511 (PMC11410197; doi:10.1371/journal.pone.0310511)
Supplement: S8 Table — ** p < .001; * p < .05. (DOCX) [file pone.0310511.s008.docx]

**S8 Table.**

| **Variables** | **T1** | | | **T2** | | |
| --- | --- | --- | --- | --- | --- | --- |
|  | **Age** | **Education** | **Gender** | **Age** | **Education** | **Gender** |
| GM | .04 | .07 | -.15** | .01 | .12* | -.11* |
| GT | .03 | .06 | -.20** | .01 | .13* | -.14* |
| AffEmp | .03 | .04 | -.35** | .04 | .02 | -.30** |
| PAS-E | .12* | .01 | -.13* | .07 | .06 | -.09* |
| PAS-I | .08 | -.02 | -.05 | .06 | .04 | -.07 |
| NFS | -.10* | -.02 | -.17** | -.12* | -.04 | -.18** |
| SS | -.07 | .01 | -.13* | -.13* | -.06 | -.18** |
| Satisfaction | .07 | .04 | -.02 | .05 | .09* | .01 |
| PastN | -.17** | -.07 | -.14* | -.17** | -.08 | -.14* |
| PresentH | -.06 | -.07 | -.07 | -.08 | -.03 | -.08 |
