# Supplementary material for: Prosocial sharing with organizations after the COVID-19 pandemic: A longitudinal test of the role of motives for helping and time perspectives
Source: PLoS One. 2024 Sep 18;19(9):e0310511. doi: 10.1371/journal.pone.0310511 (PMC11410197; doi:10.1371/journal.pone.0310511)
Supplement: S9 Table — (DOCX) [file pone.0310511.s009.docx]

**S9 Table.**

| **T2 Predicted Variable** | **T1 Predictor Variable** | ***β*** | ***p*** |
| --- | --- | --- | --- |
| GM | ***GM*** | ***.284*** | ***<.001*** |
|  | **GT** | **.184** | **<.001** |
|  | Affective Empathy | .064 | .114 |
|  | Support | .088 | .077 |
|  | Satisfaction | .017 | .738 |
|  | Past Negative | .007 | .890 |
|  | Present Hedonistic | -.060 | .156 |
| GT | GM | .066 | .182 |
|  | ***GT*** | ***.382*** | ***<.001*** |
|  | **Affective Empathy** | **.132** | **.001** |
|  | Support | -.024 | .638 |
|  | Satisfaction | .015 | .779 |
|  | **Past Negative** | **-.116** | **.018** |
|  | Present Hedonistic | .061 | .153 |
| Affective Empathy | GM | <.001 | .920 |
|  | GT | .004 | .915 |
|  | ***Affective Empathy*** | ***.696*** | ***<.001*** |
|  | Support | .065 | .091 |
|  | Satisfaction | -.043 | .280 |
|  | **Past Negative** | **.081** | **.031** |
|  | Present Hedonistic | -.013 | .689 |
| Support | GM | .000 | .992 |
|  | GT | -.021 | .597 |
|  | **Affective Empathy** | **.076** | **.021** |
|  | ***Support*** | ***.701*** | ***<.001*** |
|  | Satisfaction | .076 | .068 |
|  | Past Negative | -.033 | .397 |
|  | Present Hedonistic | .015 | .668 |
| Satisfaction | GM | .019 | .525 |
|  | GT | .004 | .896 |
|  | Affective Empathy | -.026 | .298 |
|  | **Support** | **.082** | **.008** |
|  | ***Satisfaction*** | ***.707*** | ***<.001*** |
|  | **Past Negative** | **-.122** | **<.001** |
|  | **Present Hedonistic** | **.060** | **.021** |
| Past Negative | GM | .038 | .267 |
|  | GT | .011 | .748 |
|  | Affective Empathy | .036 | .214 |
|  | Support | -.062 | .076 |
|  | **Satisfaction** | **-.071** | **.051** |
|  | ***Past Negative*** | ***.706*** | ***<.001*** |
|  | Present Hedonistic | .017 | .576 |
| Present Hedonistic | GM | .010 | .787 |
|  | GT | .024 | .520 |
|  | Affective Empathy | .000 | 1.000 |
|  | Support | .029 | .435 |
|  | **Satisfaction** | **.083** | **.030** |
|  | **Past Negative** | **.099** | **.007** |
|  | ***Present Hedonistic*** | ***.695*** | ***<.001*** |
