# Supplementary material for: Prosocial sharing with organizations after the COVID-19 pandemic: A longitudinal test of the role of motives for helping and time perspectives
Source: PLoS One. 2024 Sep 18;19(9):e0310511. doi: 10.1371/journal.pone.0310511 (PMC11410197; doi:10.1371/journal.pone.0310511)
Supplement: S10 Table — ** p < .001; * p < .05. (DOCX) [file pone.0310511.s010.docx]

**S10 Table.**

| **Variables** | **T1** | **T2** |
| --- | --- | --- |
| GM-LocalLife | .11 | .02 |
| GM-LocalEnv | .14 | .08 |
| GM-GlobalLife | .11 | -.05 |
| GM-GlobalEnv | .21 | .08 |
| GT-LocalLife | .13 | -.05 |
| GT-LocalEnv | .11 | -.01 |
| GT-GlobalLife | .09 | -.01 |
| GT-GlobalEnv | .09 | -.02 |
| AffEmp | .10* | .08 |
| PAS-E | .26* | .13 |
| PAS-I | .19* | .18 |
| NFS | -.13* | -.14* |
| SS | -.07 | -.13* |
| Satisfaction | .05 | -.03 |
| PastN | -.10* | -.05 |
| PresentH | -.03 | -.04 |
